# Development of a DNA-Based Detection Method for *Cocos Nucifera* Using TaqMan™ Real-Time PCR

**DOI:** 10.3390/foods9030332

**Published:** 2020-03-12

**Authors:** Jasmin Wrage, Oxana Kleyner, Sascha Rohn, Jürgen Kuballa

**Affiliations:** 1 GALAB Laboratories GmbH, Am Schleusengraben 7, 21029 Hamburg, Germany; jasmin.wrage@galab.de (J.W.); oxana.kleyner@galab.de (O.K.); 2 Institute of Food Chemistry, Hamburg School of Food Science, University of Hamburg, Grindelallee 117, 20146 Hamburg, Germany; rohn@chemie.uni-hamburg.de

**Keywords:** coconut allergy, *Cocos nucifera*, primer design, TaqMan™ probe, real-time PCR

## Abstract

So far, only a few cases of immunoglobulin E (IgE)-mediated coconut allergies have been described in the literature. Due to a growing consumption of coconut-containing foods in occidental countries, the number of coconut allergies may also increase. As there is no causative immunotherapy in clinical routine, appropriate food labelling is particularly important, also with regard to cross-contamination, to prevent serious health consequences. The purpose of this study was to develop a DNA-based detection method for coconut (*Cocos nucifera*). Initially, three sets of coconut-specific primers were designed and tested. A TaqMan™ probe was then developed to identify and quantify coconut by real-time PCR assay. With 27 other plant and animal species, the specificity of the primer/probe system was tested and cross reactivity was excluded. In a dilution series, a limit of detection of 1 pg/µL was determined. Thus, the developed real-time PCR assay is a suitable method to detect coconut in food.

## 1. Introduction

The coconut palm (*Cocos nucifera*) belongs to the family *Palmaceae* (*Arecaceae*) in the genus Cocos. The fruits of the coconut palm are not nuts, but drupes. The outer layer consists of a green to yellow, leathery exocarp. Below is a fibrous mesocarp, followed by a woody endocarp that surrounds the seed. This unusual fruit structure is the reason why coconuts have the ability to float thousands of kilometers between tropical islands, so that they are now spread along tropical coasts all over the world and nobody can clearly determine their center of origin. Humans have used coconuts as food, medicine, cosmetics, and as material for ropes and mats for thousands of years. In plantations, it has been cultivated since the middle of the 19th century [1]. Today, it is one of the economically most important palm species in the world, which is mainly cultivated in Indonesia, the Philippines, and India, but used all over the world [2].

In view of the growing consumption of coconut-containing products in occidental countries, the number of coconut food allergies may increase. So far, only a few cases of coconut allergies are known. But even after years of symptom-free exposure to coconuts, either through the skin or taken orally, it is still possible to develop allergenic reactions. However, not only skin rashes, but also anaphylaxis can occur, which poses a fatal danger to the affected persons. The allergic reaction is currently attributed to a 7S and an 11S globulin protein called Coc n2 and Coco n4. 7S globulins have already been described as allergens in walnuts and hazelnuts, so that some patients who are allergic to tree nuts show cross-reactions to coconuts [3,4,5,6]. As there is no causative immunotherapy in the clinical routine for food allergies so far, the only way to prevent an allergic reaction for people who are affected is to strictly avoid the allergenic food.

Consequently, appropriate food labelling is important. The EU Food Information Regulation No. 1169/2011 contains a list of 14 allergenic foods and regulates their labelling. However, this list does not yet contain coconut as an allergenic food [7]. Due to the high prevalence of peanut and tree nut allergies and concerns about possible cross-reactivity, coconut must be labelled as an ingredient in food products in the US [8]. However, special risk challenges are provided by so-called “hidden” traces of allergens, which can get into food through cross-contamination in certain food production processes. A shared concern of food allergy patients is the question, whether they can safely consume certain food products. Therefore, sensitive and specific detection methods are essential.

So far, the detection of coconut in food is routinely done using enzyme-linked immunosorbent assays (ELISA) test kits from different manufacturers. The aim of the present study was to develop a DNA-based detection method for *Cocos nucifera* using a TaqMan™ real-time PCR. Previous DNA-based detection methods have particularly focused on the detection of the 14 allergens regulated in the EU including soy, lupines, peanuts, and tree nuts [9,10,11,12]. Coconut, as a potential allergenic food, has not received much attention in the development of DNA-based detection methods yet.

For this purpose, different coconut-specific primers were designed and tested for their specificity. Subsequently, a TaqMan™ probe for real-time PCR assays was developed. In addition to its specificity, the primer/probe system was also tested for its sensitivity.

## 2. Materials and Methods

### 2.1. Sample Material

One sample of authentic coconut palm (*Cocos nucifera*) leaves were provided by Indonesian farmers, represented by Elevate Social Businesses Ltd., New York City, USA, a non-profit organization that improves the livelihoods of farmers and farming communities in Indonesia. One leaf sample each of Chilean wine palm (*Jubaea chilensis)*, oil palm (*Elaeis guineensis)*, and jelly palm (*Butia capitata)* were obtained from the Palmengarten Frankfurt/Main, Germany. All leaf samples were stored at −80 °C until DNA extraction. One coconut was purchased in a local German grocery store. One sample of coconut chips from Sri Lanka and one sample of Indonesian coconut blossom sugar were provided by a trading company (Voicevale Ltd., London, UK).

### 2.2. Sample Preparation and DNA Extraction

Palm leaves were washed with 70% ethanol, frozen in dry ice, and ground in a knife mill (Grindomix GM 200, Retsch GmbH, Haan, Germany). Coconuts were cracked and cut into small pieces with a knife. Coconut chips and coconut sugar were used directly for DNA extraction.

DNA was extracted using a cetyltrimethylammonium bromid (CTAB) method according to the German official methodology described in the German food law (LFGB), § 64 LFGB L 00.00-119:2014-02 based on DIN EN ISO 21571:2013-08 [13]. In brief, about 2 to 5 g sample were mixed with 5 to 10 mL of CTAB-containing lysis buffer and 30 µL of 20 µg/µL proteinase K. The mixture was incubated at 65 °C overnight. DNA purification was performed by a washing step with 2 mL trichloromethane, followed by DNA precipitation with CTAB precipitation buffer. The supernatant was removed and the DNA pellet dissolved in sodium chloride solution (1.2 M). Further purification with trichloromethane/3-methyl-1-butanol (24:1) and precipitation with 2-propanol and glycogen followed. The pellet was finally purified with 70% ethanol, dried, and dissolved in tris-(hydroxymethyl)-aminomethane-ethylenediaminetetraacetic-acid (TE) buffer.

### 2.3. Determination of DNA Concentration and Purity

Estimation of DNA concentration and purity was performed using a spectrophotometer (BioSpectrometer^®^ basic, Eppendorf AG, Hamburg, Germany) at 260 and 280 nm. For a quantitative determination of DNA, the fluorescence signal was measured after addition of the DNA intercalation dye PicoGreen (Quant-iT™ PicoGreen^®^ dsDNA Reagent, Thermo Fisher Scientific Inc., Waltham, MA, USA) against a λ-DNA (Thermo Fisher Scientific Inc., Waltham, MA, USA) standard curve using a microtiter plate reader (GENios, TECAN Group Ltd., Männedorf, Switzerland).

### 2.4. PCR Primers and Probes

In the present study, different sets of primers and one TaqMan™ probe were designed.

DNA sequences and descriptions of the primers and probes are listed in Table 1.

For a verification of the botanical origin of the palm leaves, universal *Palmae*-specific gene primers were designed using the DNA sequence of the nuclear gene phosphoribulokinase (*prk*). It encodes a chloroplast enzyme essential for the photosynthetic process of CO_2_ assimilation of the Calvin cycle [14] and has already been widely used in the phylogenetic analysis of *Palmae* species [15,16]. The sequences of the *prk* gene of the coconut palm (*Cocos nucifera*), Chilean wine palm (*Jubaea chilensis)*, oil palm (*Elaeis guineensis)*, and jelly palm (*Butia capitata)* were downloaded from the NCBI database and aligned with BioEdit 7.0.5 (Ibis Therapeutics Inc., Carlsbad, CA, USA) for selecting primer regions that are universal to the *Palmae* family. For the forward primer palmPRK-for, the sequence CTA GCA AAG AAT CTG ATC GAT AAG T with a length of 25 bp, a GC-content of 36%, and a melting temperature of 62 °C was selected. The sequence CAT ATT GCT TCT GTG GGT CTG with a length of 21 bp, a GC-content of 48%, and a melting temperature of 63 °C was used as reverse primer palmPRK-rev. With the web tool OligoAnalyzer (Integrated DNA Technologies Inc., Coraville, IA, USA), the formation of hairpins, self-dimers, and hetero-dimers was checked.

For the design of primer sets that could only specifically amplify *Cocos nucifera*, two different genes were used: the *prk* gene and the internal transcribed spacer 2 (*ITS2*) gene. *ITS2* is located between the coding regions of the 5.8S and the 28S rRNA of the eukaryotic nuclear ribosomal desoxyribonucleic acid (rDNA). Due to its high divergence between species, it is a commonly used marker for phylogenetic analysis across plants, also distinguishing closely related species [17,18,19].

The sequence of the *ITS2* gene of coconut palm (*Cocos nucifera*) was downloaded from the NCBI database and analyzed via PrimerBlast for appropriate primer sets. Out of 10 primer set suggestions, two were selected and tested for specificity. The primer set made up of cocosITS109-for (GGC CTC CTG AGG TAT ATC CG; length: 20 bp; GC-content: 60%; melting temperature: 67 °C) and cocosITS109-rev (CAT CCA CCA TCC ACC GTG TC; length: 20 bp; GC-content: 60%; melting temperature: 68 °C) amplifies a 109 bp long product. CocosITS197-for (TAT CCG GAT GTG GAT GCT GG; length: 20 bp; GC-content: 55%; melting temperature: 67 °C) and cocosITS197-rev (CAT CCG ATG GCT GGG GTG; length: 18 bp; GC-content: 67%; melting temperature: 69 °C) amplify a product of 197 bp length.

For the primer set of the *prk* gene, the coconut-specific sequences cocosPRK-for (ACA AGA CCT ACT GGA CTG G; length: 19 bp; GC-content: 53%; melting temperature: 63 °C) and cocosPRK-rev (TCT GAT ATG TAT AAG ACT CAG CA; length: 23 bp; GC-content: 35%; melting temperature: 59 °C), which amplify a 164 bp long product, were selected based on the alignment in BioEdit.

The synthesis of the PCR primers was purchased from Integrated DNA Technologies Inc. (Coraville, IA, USA).

The designed TaqMan™ probe was purchased from TIB MOLBIOL Syntheselabor GmbH (Berlin, Germany).

### 2.5. Conventional PCR

Conventional polymerase chain reaction (PCR) was performed using a commercially available master mix (Q5^®^ Hot Start High-Fidelity Master Mix (2X), New England BioLabs, Inc., Ipswich, MA, USA). The amount of reaction components and the thermocycling conditions were selected according to manufacturer’s instructions. For a total volume of 25 µL PCR reaction mixture, 1.25 µL of forward primer (10 µM), 1.25 µL of reverse primer (10 µM), 5 µL of DNA template (1–5 ng/µL), and 5 µL of distilled water were added to 12.5 µL of master mix. Non-template samples with 5 µL of distilled water instead of DNA were used as negative controls.

The PCR was run on a thermocycler (Mastercycler^®^ nexus gradient, Eppendorf AG, Hamburg, Germany) with the following conditions: initial denaturation at 98 °C for 10 min followed by 35 cycles of denaturation at 98 °C for 10 s, annealing at a primer specific temperature (euk18S: 68 °C, palmPRK: 63 °C, cocosPRK: 60 °C, cocosITS109: 68 °C, cocosITS197: 68 °C) for 25 s, and extension at 72 °C for 15 s and 1 cycle of final extension at 72 °C for 2 min.

### 2.6. Gel Electrophoresis

All PCR products were separated with gel electrophoresis on a 2% agarose gel containing 5 µL of the intercalating nucleic acid stain GelRed^®^ 10000x (Biotium, Inc., Fremont, CA, USA) in 1x tris-(hydroxymethyl)-aminomethane-borate-ethylenediaminetetraacetic-acid (TBE) buffer. Then, 4 µL PCR product was mixed with 3 µL DNA loading dye (FastDigest Green Buffer (10x), Thermo Fisher Scientific Inc., Waltham, MA, USA), loaded into each well, and electrophoresed at 90 V for 1.5 h. Electrophoresis gels were visualized and analyzed by UV transillumination. A 100 bp DNA ladder (Quick-Load^®^ Purple 100 bp DNA Ladder, New England BioLabs, Inc., Ipswich, MA, USA) was used for comparison.

### 2.7. DNA Sequencing

To confirm the sequence of PCR products, the DNA was isolated from the agarose gel using a PCR clean-up and gel extraction kit (NucleoSpin^®^ Gel and PCR Clean-up, MACHEREY-NAGEL GmbH & Co. KG, Düren, Germany) and was sent to a laboratory specialized in sequencing (Eurofins Genomics Germany GmbH, Cologne, Germany). The results were verified using a sequence comparison with BLASTn against the NCBI nucleotide database.

### 2.8. Real-Time PCR

Real-time PCR was run on a qTOWER^3^-thermocycler (Analytik Jena AG, Jena, Germany). Each reaction mixture of 20 µL contained 10 µL of innuMIX qPCR MasterMix Probe (Analytik Jena AG, Jena, Germany), 3.8 µL of distilled water, 0.5 µL of forward primer (20 µM), 0.5 µL of reverse primer (20 µM), 0.2 µL of probe (20 µM), and 5 µL of DNA template (10 ng/µL). The thermocycling conditions were set as follows: 50 °C for 2 min, 95 °C for 10 min, followed by 45 cycles of 95 °C for 15 s, and 60 °C for 1 min.

#### 2.8.1. Specificity of the Real-Time PCR Assay

In order to verify specificity, the cross-reactivity of the primer/probe system was tested with DNA isolated from the following plant and animal species: almond (*Amygdalus communis*), apricot (*Armeniaca vulgaris*), Atlantic redfish (*Sebastes norvegicus*), brazil nut (*Bertholletia excelsa*), cattle (*Bos taurus*), celery (*Apium graveolens*), chicken (*Gallus gallus*), Chilean wine palm (*Jubaea chilensis*), chili pepper (*Capsicum frutescens*), hazelnut (*Corylus avellana*), lupine (*Lupine* spp.), mango (*Mangifera indica*), oil palm (*Elaeis guineensis*), pangasius (*Pangasianodon hypophthalmus*), papaya (*Carica papaya*), peanut (*Arachis hypogaea*), pepper (*Capsicum annuum*), pistachio (*Pistacia vera*), rye (*Secale cereale*), sesame (*Sesamum indicum*), soy (*Glycine max*), sunflower (*Helianthus annuus*), turkey (*Meleagris gallopavo*), walnut (*Juglans regia*), wheat (*Triticum aestivum*), white mustard (*Sinapis alba*), jelly palm (*Butia capitata*).

For evaluating sensitivity of the real-time PCR assay, the isolated targeted DNA was serially diluted 10-fold, reaching a concentration range from 100 ng/µL to 0.1 pg/µL.

## 3. Results

### 3.1. Extraction of DNA from Palm Leaves

DNA was isolated from coconut leaves, Chilean wine, oil, and jelly palm in four replicates (A, B, C, D) using the standard CTAB method. The purity and concentration of the DNA was determined spectrophotometrically. Performing a conventional PCR, followed by gel electrophoresis of the PCR products, the amplifiability of the isolated DNA was verified. Therefore, the well-established primers euk18S-for and euk18S-rev were used to amplify a 137 bp long sequence of the 18S ribosomal ribonucleic acid (rRNA) gene specific for eukaryotes [20,21]. The results of DNA isolation and the conventional PCR assay are presented in Table 2. DNA was successfully isolated from all samples and of comparatively good quality.

### 3.2. Verification of the Botanical Origin of the Palm Leaves

For a verification of the botanical origin of the palm leaves, universal *Palmae*-specific gene primers palmPRK-for and palmPRK-rev were designed and a conventional PCR followed by a gel electrophoretic analysis was performed. The PCR products, varying in length according to the palm species, were sequenced and verified with a Blastn search in the NCBI nucleotide database. The result of the sequence comparison clearly matched with *Cocos nucifera*, *Jubaea chilensis*, *Elaeis guineensis*, and *Butia capitata* and confirmed the botanical origin of the palm leaves.

### 3.3. Design and Specificity of Coconut-Specific Primers

Three different sets of primers, cocosPRK, cocosITS109, and cocosITS197, which should only specifically amplify *Cocos nucifera*, were designed.

**Figure 1:**
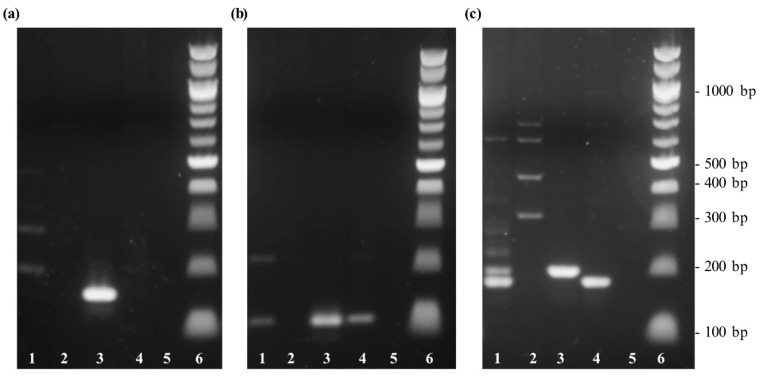
Gel electrophoresis of DNA extracted from different palm species to test the specificity of different sets of primer. Lane 1: Chilean wine palm (*Jubaea chilensis*); lane 2: oil palm (*Elaeis guineensis*); lane 3: coconut palm (*Cocos nucifera*), lane 4: jelly palm (*Butia capitata*); lane 5: non-template sample with water; lane 6: 100 bp DNA ladder. (**a**) PCR amplification with the primer set cocosPRK. (**b**) PCR amplification using the primer set cocosITS109. (**c**) PCR amplification performed with the primer set cocosITS197.

In order to verify the specificity of the primer sets, a conventional PCR, followed by a gel electrophoretic analysis was performed using DNA templates of coconut palm (*Cocos nucifera*), Chilean wine palm (*Jubaea chilensis*), oil palm (*Elaeis guineensis*), and jelly palm (*Butia capitata*). The results are presented in Figure 1. While the primer set cocosPRK only showed PCR products in a length of 164 bp specific for *Cocos nucifera*, the primer sets cocosITS109 and cocosITS197 also amplified other species.

The coconut-specific primer set of the *prk* gene was selected for follow-up experiments.

### 3.4. PCR Amplification of DNA from Coconut, Coconut Chips, and Coconut Blossom Sugar

**Figure 2:**
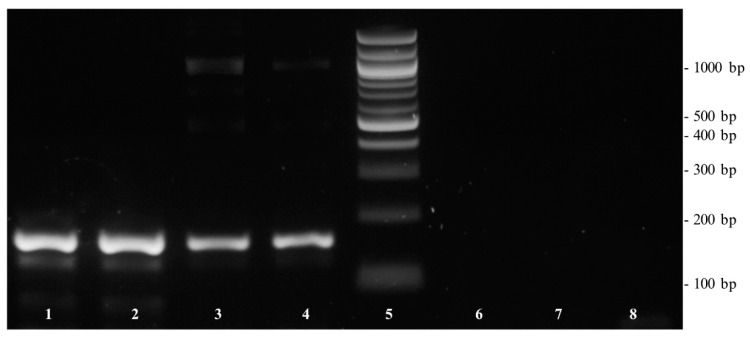
Gel electrophoresis of DNA extracted from coconut, coconut chips, and coconut blossom sugar. Lane 1–2: coconut chips; lane 3–4: coconut; lane 5: 100 bp DNA ladder; lane 6–7: coconut blossom sugar; lane 8: non-template sample with water.

For further verification of the cocosPRK primer set, a conventional PCR followed by gel electrophoresis was performed using DNA isolated from coconut, coconut chips, and coconut blossom sugar. As shown in Figure 2, positive results were obtained with the coconut and coconut chips samples. Negative PCR results were obtained using the DNA template isolated from coconut blossom sugar. As the concentration of DNA isolated from coconut blossom sugar determined by spectrophotometer was 210 ng/µL and the quotient of A260/A280 was 1.95, the negative amplification reaction was not the result of insufficient or poor DNA quality. Amplifiability of the DNA with the eukaryotic-specific primers euk18S confirmed that there were no PCR inhibitory contaminants. However, as coconut sap is a fast-fermenting product [22,23], there is the hypothesis that the isolated DNA was from yeasts and coconut-specific DNA was too low in concentration and quality for PCR amplification.

### 3.5. Development of a TaqMan™ Real-Time PCR Assay

For the development of a coconut-specific real-time PCR assay, a TaqMan™ probe was developed. TaqMan™ probes are hydrolysis probes which are oligonucleotides consisting of a reporter fluorophore attached to the 5’-end, a quencher at the 3’-end, and a 3′-extension block. They are located within the gene fragment delimited by the primer set. During amplification, the probe is detached from the DNA template and the fluorophore and quencher separate so that the fluorescence signal emitted by the fluorophore is no longer quenched [24,25].

The TaqMan™ probe was placed inside the gene fragment, which was enclosed by the cocosPRK primer set. As the gene fragment had many regions with homopolymer repeats and a very low GC-content, there were not many options to place the probe. A 30 bp long sequence (AAT TGT CTC ATT ATC TCA ATG AAC CGG GTG; GC-content: 40%; melting temperature: 68 °C) in close proximity to the 3’-end of the reverse primer was selected. Using the OligoAnalyzer web tool (Integrated DNA Technologies Inc., Coraville, IA, USA), the formation of hairpins, self-dimers, and hetero-dimers was checked. As fluorophore 6-carboxyfluorescein (6-FAM) with an absorption wavelength of 495 nm and an emission wavelength of 520 nm was used. It is a long-established and widely used fluorescent dye in life sciences [26,27]. The BlackHole™ Dark Quencher BHQ-1 from LGC Biosearch Technologies Inc. (London, UK) was used as quencher. It quenches emission at wavelengths of 480–580 nm and has the advantage that it does not release any additional fluorescence compared to the previously used tetramethylrhodamine (TAMRA) quencher [28,29]. Synthesis of the probe was purchased from TIB MOLBIOL Syntheselabor GmbH (Berlin, Germany).

### 3.6. Assay Specificity and Sensitivity

**Figure 3:**
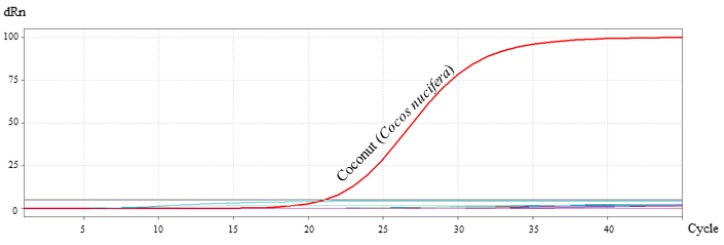
Specificity test of the primer/probe system for coconut identification. No amplification was detected with species other than coconut.

The specificity of the primer/probe system was tested by amplification of DNA in a concentration of 10 ng/µL from 28 different plant and animal species mentioned in Section 2.8.1. As shown in Figure 3, no amplification was detected with species other than *Cocos nucifera*. The result showed that the primer/probe system was specific for the identification of *Cocos nucifera* and showed no cross-reactivity.

**Figure 4:**
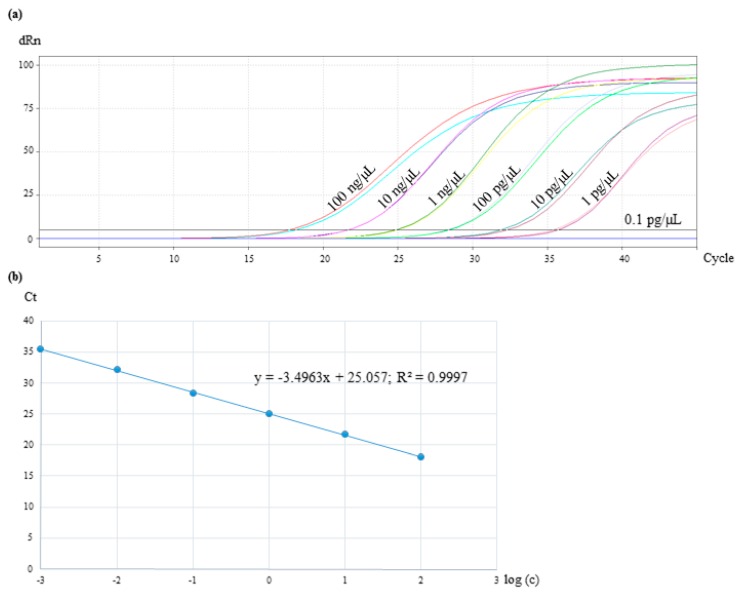
Sensitivity test of the real-time PCR assay with 10-fold diluted DNA of coconut in a concentration range from 100 ng/µL to 0.1 pg/µL. The reactions were performed in duplicates. (**a**) The amplification plot shows C_t_ values for samples from 100 ng/µL to 1 pg/µL. (**b**) Standard curve of the coconut TagMan real-time PCR system by plotting C_t_ values against the log_10_ input DNA concentration.

For the sensitivity test of the real-time PCR assay, the DNA of *Cocos nucifera* was serially diluted 10-fold for reaching a concentration range from 100 ng/µL to 0.1 pg/µL. The reactions were performed in duplicates. The results presented in Figure 4a showed that samples with a DNA concentration of 0.1 pg/µL were not amplified. The lowest quantifiable level of *Cocos nucifera* and consequently, the limit of detection, was 1 pg/µL with a C_t_ value of 35. By plotting C_t_ values against the log_10_ input DNA concentration, a standard curve was constructed (Figure 4b). Ideally, a real-time PCR reaction should have an efficiency in a range of 90% to 110%, which corresponds to a slope between −3.58 and −3.10 [30]. With −3.49, it was in the optimal range in the present study.

## 4. Discussion

In the present study, three different sets of primers were designed and tested for their specificity towards *Cocos nucifera*. While the primer sets cocosITS107 and cocosITS197 amplify PCR products with very similar base length also for other palm species, cocosPRK amplifies a 164 bp long amplicon characteristic for *Cocos nucifera*. Matching this gene fragment, a TaqMan™ probe was developed. The primer/probe system showed no cross-reactivity to other plant and animal species and was therefore highly specific to *Cocos nucifera*. The developed real-time PCR assay was sensitive to detect DNA concentrations of 1 pg/µL and can be used to detect traces of coconut. Unfortunately, DNA isolated from coconut blossom sugar was not amplifiable with the coconut-specific primers cocosPRK. For this purpose, extraction methods other than the CTAB method should be developed and evaluated.

The developed real-time PCR assay is an alternative method to the existing ELISA test kits. The present results can contribute to detect coconut in food and to protect consumers from hidden traces and to prevent dangerous food allergic reactions. Instead of ELISA, PCR is an indirect method for the detection of food allergens. While ELISA is based on highly specific antigen–antibody reactions and detects allergenic proteins, real-time PCR detects and quantifies matrix-specific DNA sequences [31]. A detection of DNA of allergenic food does not reveal anything about the content of allergenic traces in the form of allergenic proteins. It does not consider the actual allergen content and the potential of an allergenic response. But for the comparison of the two methods, it must be taken into account that ELISA also detects only a limited number of target proteins, considering that 17 peanut allergens have been described, and while DNA often breaks down into small sequences of less than 200 bp, proteins are often denatured, which in turn can influence the allergenic response [32,33,34]. However, the mandatory need of animals for producing antibodies is one of the main disadvantages of ELISA, because the production procedure of antibodies is rather cruel for the animal and therefore getting more and more restricted in many countries, e.g., by the European legislation (directive 2010/63/EU) [35].

Regardless of the actual allergenic content, many studies showed that ELISA and real-time PCR assays achieve comparable results and that real-time PCR is therefore an important tool for the rapid detection of allergenic food traces in the food industry [12,36].

## Figures and Tables

**Table 1:** DNA sequences and description of the forward (= for.) and reverse (= rev.) primers and probes listed alphabetically.

Primer/Probe Name	Description	Target Gene	Sequence (5′ → 3′)
**euk18S-for.**	Eukaryotic-specific for. primer	18SrRNA	TCT GCC CTA TCA ACT TTC GAT GGT A
**euk18S-rev.**	Eukaryotic-specific rev. primer	18SrRNA	AAT TTG CGC GCC TGC TGC CTT CCT T
**cocosITS109-for.**	Coconut-specific for. primer	ITS	GGC CTC CTG AGG TAT ATC CG
**cocosITS109-rev.**	Coconut-specific rev. primer	ITS	CAT CCA CCA TCC ACC GTG TC
**cocosITS197-for.**	Coconut-specific for. primer	ITS	TAT CCG GAT GTG GAT GCT GG
**cocosITS197-rev.**	Coconut-specific rev. primer	ITS	CAT CCG ATG GCT GGG GTG
**cocosPRK-for.**	Coconut-specific for. primer	prk	ACA AGA CCT ACT GGA CTG G
**cocos-PRK-rev.**	Coconut-specific rev. primer	prk	TCT GAT ATG TAT AAG ACT CAG CA
**cocosPRK**	Coconut probe	prk	FAM-AAT TGT CTC ATT ATC TCA ATG AAC CGG GTG-BHQ1
**palmPRK-for.**	Palmae-specific for. primer	prk	CTA GCA AAG AAT CTG ATC GAT AAG T
**palmPRK-rev.**	Palmae-specific rev. primer	prk	CAT ATT GCT TCT GTG GGT CTG

**Table 2:** Properties of the DNA extracted from the leaves of coconut palm (*Cocos nucifera*), Chilean wine palm (*Jubaea chilensis*), oil palm (*Elaeis guineensis*), and jelly palm (*Butia capitata*).

*Palmea* Species	Sample	DNA Concentration [µg/µL]	A260/A280	Amplifiability
Chilean wine palm (*Jubaea chilensis*)	A B C D	1.64 2.21 1.60 1.47	2.00 2.07 2.01 2.01	+
Coconut palm (*Cocos nucifera*)	A B C B	1.88 1.86 1.86 1.77	1.98 1.92 1.92 1.97	+
Jelly palm (*Butia capitata*)	A B C D	2.71 2.80 2.35 2.49	1.94 1.94 1.93 1.93	+
Oil palm (*Elaeis guineensis*)	A B C D	2.73 2.98 2.60 1.91	1.97 1.97 1.98 2.01	+
